# A Variable Precision Attribute Reduction Approach in Multilabel Decision Tables

**DOI:** 10.1155/2014/359626

**Published:** 2014-08-06

**Authors:** Hua Li, Deyu Li, Yanhui Zhai, Suge Wang, Jing Zhang

**Affiliations:** ^1^Key Laboratory of Computational Intelligence and Chinese Information Processing of Ministry of Education, Shanxi University, Taiyuan, Shanxi 030006, China; ^2^Department of Mathematics and Physics, Shijiazhuang Tiedao University, Shijiazhuang, Hebei 050043, China; ^3^School of Computer and Information Technology, Shanxi University, Taiyuan, Shanxi 030006, China

## Abstract

Owing to the high dimensionality of multilabel data, feature selection in multilabel learning will be necessary in order to reduce the redundant features and improve the performance of multilabel classification. Rough set theory, as a valid mathematical tool for data analysis, has been widely applied to feature selection (also called attribute reduction). In this study, we propose a variable precision attribute reduct for multilabel data based on rough set theory, called *δ*-confidence reduct, which can correctly capture the uncertainty implied among labels. Furthermore, judgement theory and discernibility matrix associated with *δ*-confidence reduct are also introduced, from which we can obtain the approach to knowledge reduction in multilabel decision tables.

## 1. Introduction

Conventional supervised learning deals with the single-label data, where each instance is associated with a single class label. However, in many real-world tasks, one instance may simultaneously belong to multiple class lultilabel decision tablabels, for example, in text categorization problems, where every document may be labeled as several predefined topics, such as religion and political topics [1]; in image annotation problems, a photograph may be associated with more than one tag, such as elephant, jungle, and Africa [2]; in functional genomics, each gene may be related to a set of functional classes, such as metabolism, transcription, and protein synthesis [3]. Such data are called multilabel data.

Owing to the high dimensionality of multilabel data, feature selection in multilabel learning will be necessary in order to reduce the redundant features and improve the performance of multilabel classification. Among various feature selection approaches, rough set theory, proposed by Pawlak [4], has attracted much attention due to its special advantage, that is, the capability of studying imprecise, incomplete, or vague information without requiring prior information.

Feature selection in rough set theory is also called attribute reduction. Generally speaking, attribute reduction can be interpreted as a process of finding the minimal set of attributes that can preserve or improve one or several criteria. The minimal set of attributes is called an attribute reduct. In past few years, many researchers have done much work on attribute reduction and the summarization of important results has been done in [5, 6]. The idea of attribute reduction using positive region was first originated in [7, 8], aiming to remove redundant attributes as much as possible while retaining the so-called positive regions. Afterwards, Ziarko introduced the variable precision rough set model and *β*-reduct to improve the ability of modeling uncertain information [9]. Furthermore, Kryszkiewicz proposed five kinds of attribute reducts for inconsistent information systems [10] and the relationships in these five reducts and some related results are reconsidered and rectified in [11]. Applying discernibility matrix, Skowron and Rauszer [12] proposed an attribute reduction algorithm by computing disjunctive normal form, which is able to obtain all attribute reducts of a given information system. On the other hand, for obtaining a single reduct from a given information system in a relatively short time, many heuristic attribute reduction algorithms have been developed. In order to reduce computational time, Xu et al. [13] proposed a quick attribute reduction algorithm with complexity of max(*O*(|*C*||*U*|), *O*(|*C*|^2^|*U*/*C*|)). Further, Qian et al. [14] developed a common accelerator based on four kinds of heuristic reduction algorithms to improve the time efficiency of a heuristic search process.

As far as we know, however, little work has been done on applying rough set theory to feature selection in multilabel learning. Although directly applying the existing attribute reduction methods to multilabel data is possible, it does not take into account the uncertainty conveyed by labels and thus can be enhanced further. In this paper, we propose a new attribute reduct for multilabel data, namely, *δ*-confidence reduct, which overcomes the limitations of existing attribute reduction methods to multilabel data. Furthermore, judgement theory and discernibility matrix associated with *δ*-confidence reduct are also established. These results provide approaches to knowledge reduction for multilabel data, which are significant in both the theoretic and applied perspectives.

The rest of this paper is organized as follows. Some basic notions in rough set theory are briefly reviewed in Section 2.  Section 3 is devoted to introducing multilabel decision table and analyzing the limitations of the existing attribute reduction methods to multilabel data. In Section 4, the new attribute reduct, *δ*-confidence reduct, is proposed and the corresponding judgement theorem and discernibility matrix are also introduced. A computative example is also given to illustrate our approaches. Finally, in Section 5, we conclude the paper with a summary and outlook for further research.

## 2. Preliminaries

In this section, we will review several basic concepts in rough set theory.

A decision table is an information system *S* = (*U*, *A* ∪ *D*) with *A*∩*D* = *∅*, where *U* = {*x*_1_, *x*_2_,…, *x*_*n*_} is a nonempty, finite set of objects called universe; *A* = {*a*_1_, *a*_2_,…, *a*_*p*_} is a nonempty, finite set of condition attributes; *D* = {*d*_1_, *d*_2_,…, *d*_*q*_} is a nonempty, finite set of decision attributes. Each nonempty subset *B*⊆*A* determines an indiscernibility relation in the following way:

(1) $$\begin{array}{c} R_{B}=\left\{\left(x,y\right)\in U\times U:a\left(x\right)=a\left(y\right),\forall a\in B\right\}. \end{array}$$

The indiscernibility relation *R*_*B*_ partitions *U* into some equivalence classes given by *U*/*R*_*B*_ = {[*x*]_*B*_ : *x* ∈ *U*}, where [*x*]_*B*_ denotes the equivalence class determined by *x* with respect to *B*; that is,

(2) $$\begin{array}{c} {\left[x\right]}_{B}=\left\{y\in U:\left(x,y\right)\in R_{B}\right\}. \end{array}$$

Let *X*⊆*U* and *B*⊆*A*. One can define a lower approximation of *X* and an upper approximation of *X* by

(3) RB_(X)={x∈U:[x]B⊆X}=⋃{[x]B:[x]B⊆X},

(4) RB¯(X)={x∈U:[x]B∩X≠∅}=⋃{[x]B:[x]B∩X≠∅},

respectively. The lower approximation is called the positive region of *X* and denoted alternatively as POS_*B*_(*X*). If $\underline{R_{B}}(X)\neq \bar{R_{B}}(X)$, then *X* is called a rough set.

Attribute reduct is one of the most important topics in rough set theory, which aims to delete the irrelevant or redundant attributes while retaining the discernible ability of original attributes. Among many attribute reduction methods, the positive region reduct [7, 8] is a representative method.

**Definition 1 .**

Let *S* = (*U*, *A* ∪ *D*) be a decision table and *B*⊆*A*. *B* is a positive region reduct of *S* if and only if *B* satisfies the following conditions:

1. POS_*B*_(*D*) = POS_*A*_(*D*),
2. POS_*B*′_(*D*) ≠ POS_*A*_(*D*) for any *B*′⊆*B*,

where POS_*B*_(*D*) = ⋃_*i*=1_^*r*^POS_*B*_(*D*_*i*_) and *D*_*i*_  (*i* = 1,2,…, *r*) are the equivalence classes, called decision classes generated by the indiscernibility relation *R*_*D*_ = {(*x*, *y*) ∈ *U* × *U* : *d*(*x*) = *d*(*y*), ∀*d* ∈ *D*}.

## 3. The Multilabel Data

In this section, we first introduce the multilabel decision table and then analyze the limitations of existing attribute reduction approaches to multilabel data.

### 3.1. The Multilabel Data

Multilabel data can be characterized by a multilabel decision table *S* = (*U*, *A*, *F*, *L*, *G*) with *A*∩*L* = *∅*, where *U* = {*x*_1_, *x*_2_,…, *x*_*n*_} is a nonempty finite set of objects, called universe; *A* = {*a*_1_, *a*_2_,…, *a*_*p*_} is a nonempty finite set of condition attributes, called condition attribute set; *F* = {*a*_*k*_ : *U* → *V*_*k*_, *k* = 1,2,…, *p*} is a set of information functions with respect to condition attributes and *V*_*k*_ is the domain of *a*_*k*_; *L* = {*l*_1_, *l*_2_,…, *l*_*q*_} is a nonempty finite set of *q* possible labels called label set; *G* = {*l*_*k*_ : *U* → *V*_*k*_′, *k* = 1,2,…, *q*} is a set of information functions with respect to labels and *V*_*k*_′ = {0,1} is the domain of the label *l*_*k*_. If the object *x* is associated with label *l*_*k*_, then *l*_*k*_(*x*) = 1; otherwise *l*_*k*_(*x*) = 0. The 5-tuple (*U*, *A*, *F*, *L*, *G*) can be expressed more simply as (*U*, *A*, *L*) if *F* and *G* are understood.

Some conventions in multilabel learning are shown as follows.

1. The object having no labels is irrelevant to multilabel learning and thus is not taken into account in the setting [15, 16]. Note that this convention is a prerequisite for the proposed approach, as discussed in Section 4.
2. Each label from *L* associates with at least one object in *U* [17].

The following example depicts a multilabel decision table in more detail.

**Example 2 .**

A multilabel decision table *S* = (*U*, *A*, *L*) is presented in Table 1, which is a part of document topic classification problem. It consists of nine documents that belong to one or more of three labels: religion, science, and politics. It can be seen that *U* = {*x*_1_, *x*_2_, *x*_3_, *x*_4_, *x*_5_, *x*_6_, *x*_7_, *x*_8_, *x*_9_}, *A* = {*a*, *b*, *c*}, and *L* = {*l*_1_, *l*_2_, *l*_3_}. Note that each object in *U* is associated with at least one label from *L* and each label from *L* is associated with at least one object in *U*.

### 3.2. The Limitations of Existing Attribute Reduction Approaches to Multilabel Data

In this section, we mainly analyze the limitations of existing attribute reduction approaches to multilabel data.

For a multilabel decision table *S* = (*U*, *A*, *L*), each label attribute can be viewed as a binary decision attribute and then form an indiscernibility relation *R*_*L*_ as follows:

(5) $$\begin{array}{c} R_{L}=\left\{\left(x,y\right)\in U\times U:l\left(x\right)=l\left(y\right),\forall l\in L\right\}. \end{array}$$

*R*_*L*_ partitions *U* into a family of equivalence classes given by *U*/*R*_*L*_ = {*D*_1_, *D*_2_,…, *D*_*r*_}. In this case, most existing attribute reduction approaches can be directly applied to multilabel data. Here we consider, for instance, positive region reduct, to delete redundant condition attributes in multilabel decision tables. The following example illustrates this process.

**Example 3 .**

For the multilabel decision table *S* = (*U*, *A*, *L*) given by Table 1, we can conclude that

(6) URA={X1,X2,X3,X4,X5}={{x1,x3},{x2},{x4,x5,x7},{x6}{x8,x9}};URL={D1,D2,D3,D4,D5}={{x1,x2},{x3},{x4,x6}{x5,x8},{x7,x9}}.

Thus, we have POS_*A*_(*D*) = {*x*_2_, *x*_6_} = *X*_2_ ∪ *X*_4_. It means that the other equivalence classes *X*_1_, *X*_3_, and *X*_5_ in *U*/*R*_*A*_ are all uncertain with respect to the label set *L*. For instance, consider the equivalence class *X*_1_ = {*x*_1_, *x*_3_}. Notice that *x*_1_ and *x*_3_ are indiscernible with respect to *A* while their respect label sets {*l*_1_} and {*l*_1_, *l*_2_} are discernible with respect to *L*. This means *X*_1_ is uncertain with respect to the label set *L*. Furthermore, we can calculate that

(7) UR{a,c}={Y1,Y2,Y3,Y4}={X1∪X5,X2,X3,X4}={{x1,x3,x8,x9},{x2},{x4,x5,x7},{x6}};UR{b,c}={Z1,Z2,Z3,Z4}={X1,X2,X3∪X5,X4}={{x1,x3},{x2},{x4,x5,x7,x8,x9},{x6}}.

Since *X*_1_, *X*_3_, and *X*_5_ are all uncertain with respect to *L*, they can be safely merged without any information loss. In other words, removing the attribute *b* or *a* is valid from the perspective of rough sets. Moreover, one can check that no more attributes can be removed from either {*a*, *c*} or {*b*, *c*}; so {*a*, *c*} and {*b*, *c*} are both positive region reducts.

However, notice that all objects in *X*_1_ must be associated with label *l*_1_ and may be associated with label *l*_2_ in the probability of 1/2 and must not be associated with label *l*_3_, whereas all objects in *X*_5_ must not be associated with label *l*_1_ and must be associated with label *l*_2_ and may be associated with label *l*_3_ in the probability of 1/2. Thus, the uncertainty of *X*_1_ and *X*_5_ is different, and the equivalence class *Y*_1_, the union of *X*_1_ and *X*_5_, cannot preserve the uncertainty conveyed by labels. This implies that {*a*, *c*} is not an appropriate attribute reduct. Similarly, {*b*, *c*} is also not an appropriate attribute reduct for multilabel data.

Through the above analysis, we know that some positive region reducts cannot preserve uncertainty implied among labels for multilabel data. In fact, since the computation of positive region reduct has to refer to the indiscernibility relation *R*_*L*_, the uncertainty conveyed by labels may be not analyzed thoroughly. Furthermore, note that the uncertainty characterized by *R*_*L*_ is also considered by the other existing attribute reduction methods; so they have the same limitations for multilabel data like positive region reduct. Thus it is necessary to reconsider attribute reduction method for multilabel data.

## 4. The New Attribute Reduction Approach in Multilabel Data Decision Tables

In this section, we will introduce a new attribute reduct referred to as *δ*-confidence reduct and show some advantages of *δ*-confidence reduct in unraveling the uncertainty of multilabel data. Moreover, judgement theory and discernibility matrix associated with *δ*-confidence reduct are also established.

### 4.1. *δ*-Confidence Reduct in Multilabel Decision Tables

First, we present the following definition.

**Definition 4 .**

Let *S* = (*U*, *A*, *L*) be a multilabel decision table, where *A* = {*a*_1_, *a*_2_,…, *a*_*p*_} and *L* = {*l*_1_, *l*_2_,…, *l*_*q*_}. For each label *l*_*i*_, one defines the label decision set as the collection of all possible objects having the label:

(8) $$\begin{array}{c} H_{i}=\left\{x\in U:l_{i}\left(x\right)=1\right\}. \end{array}$$

Considering Convention 1 of multilabel learning, one has ∪_*i*=1_^*q*^ *H*_*i*_ = *U*; that is, *H*_1_, *H*_2_,…, *H*_*q*_ form a cover of *U*.

In the following, we present a particular function to characterize the uncertainty implied among labels.

**Definition 5 .**

Let *S* = (*U*, *A*, *L*) be a multilabel decision table, let *P*(*L*) be the power set of label set *L*, and let *H*_1_, *H*_2_,…, *H*_*q*_ be *q* label decision sets. Given a subset *B*⊆*A* and *δ* ∈ [0,1], one defines a *δ*-confidence label function *η*_*B*_^*δ*^ : *U* → *P*(*L*), as follows:

(9) $$\begin{array}{c} \eta_{B}^{\delta }\left(x\right)=\{\begin{array}{cc} \left\{l_{i}\in L:\frac{\left|{\left[x\right]}_{B}\cap H_{i}\right|}{\left|{\left[x\right]}_{B}\right|}\geq \delta \right\} & \text{if}\;\;0<\delta \leq 1;\\ \left\{l_{i}\in L:\frac{\left|{\left[x\right]}_{B}\cap H_{i}\right|}{\left|{\left[x\right]}_{B}\right|}>0\right\} & \text{if}\;\;\delta =0. \end{array} \end{array}$$

The *δ*-confidence label function *η*_*B*_^*δ*^(*x*) is the collection of the labels that associate with at least *δ*% objects in [*x*]_*B*_. In other words, *η*_*B*_^*δ*^(*x*) is the collection of the labels which associate with each object in [*x*]_*B*_ by at least *δ*% confidence level.

**Example 6 .**

Consider the multilabel decision table *S* = (*U*, *A*, *L*) given by Table 1. If *δ* = 0.6, then the 0.6-confidence label function with respect to attribute set A can be calculated that

(10) ηA0.6(x1)=ηA0.6(x3)={l1},ηA0.6(x2)={l1},ηA0.6(x4)=ηA0.6(x5)=ηA0.6(x7)={l2,l3},ηA0.6(x6)={l1,l2,l3},ηA0.6(x8)=ηA0.6(x9)={l2}.

We have the following property.

**Theorem 7 .**

Let *S* = (*U*, *A*, *L*) be a multilabel decision table, *B*, *C*⊆*A*. Then

1. if 0 ≤ *δ*_1_ ≤ *δ*_2_ ≤ 1, then *η*_*B*_^*δ*_2_^(*x*)⊆*η*_*B*_^*δ*_1_^(*x*);
2. if *B*⊆*C*, then *η*_*B*_^1^(*x*)⊆*η*_*C*_^1^(*x*)⊆*η*_*C*_^0^(*x*)⊆*η*_*B*_^0^(*x*);
3. for any *x* ∈ *U*, *η*_*B*_^0^(*x*) ≠ *∅*;
4. if [*x*]_*B*_ = [*y*]_*B*_, then *η*_*B*_^*δ*^(*x*) = *η*_*B*_^*δ*^(*y*).

**Proof**

(1) Let *l*_*i*_ ∈ *η*_*B*_^*δ*_2_^(*x*). Then we have |[*x*]_*B*_∩*H*_*i*_|/[*x*]_*B*_ ≥ *δ*_2_. Note that *δ*_2_ ≥ *δ*_1_; thus |[*x*]_*B*_∩*H*_*i*_|/[*x*]_*B*_ ≥ *δ*_1_. It means that *l*_*i*_ ∈ *η*_*B*_^*δ*_1_^(*x*). Therefore *η*_*B*_^*δ*_2_^(*x*)⊆*η*_*B*_^*δ*_1_^(*x*).

(2) Since *B*⊆*C*, we have [*x*]_*C*_⊆[*x*]_*B*_.

If *l*_*i*_ ∈ *η*_*B*_^1^(*x*), then [*x*]_*B*_⊆*H*_*i*_. Since [*x*]_*C*_⊆[*x*]_*B*_, we have [*x*]_*C*_⊆*H*_*i*_. Thus *l*_*i*_ ∈ *η*_*C*_^1^(*x*). Therefore *η*_*B*_^1^(*x*)⊆*η*_*C*_^1^(*x*).

According to Theorem 7 (1), we have *η*_*C*_^1^(*x*)⊆*η*_*C*_^0^(*x*).

If *l*_*j*_ ∈ *η*_*C*_^0^(*x*), then [*x*]_*C*_∩*H*_*j*_ ≠ *∅*. Since [*x*]_*C*_⊆[*x*]_*B*_, we have [*x*]_*B*_∩*H*_*j*_ ≠ *∅*. That means *l*_*j*_ ∈ *η*_*B*_^0^(*x*). Therefore *η*_*C*_^0^(*x*)⊆*η*_*B*_^0^(*x*).

(3) If there exists *x* ∈ *U* such that *η*_*B*_^0^(*x*) = *∅*, then [*x*]_*B*_∩*H*_*i*_ = *∅*,  *i* = 1,2,…, *q*. Thus [*x*]_*B*_∩(*H*_1_ ∪ *H*_2_ ∪ ⋯*H*_*q*_) = *∅*. Since ∪_*i*=1_^*q*^ *H*_*i*_ = *U*, we have [*x*]_*B*_∩(*H*_1_ ∪ *H*_2_ ∪ ⋯*H*_*q*_) = [*x*]_*B*_∩*U* ≠ *∅*. It is a contradiction.

(4) It is straightforward by the definition of *η*_*B*_^*δ*^(*x*) and [*x*_*B*_] = [*y*]_*B*_.

Now we define the consistent *δ*-confidence set using *δ*-confidence label function. Furthermore, we present the definition of new attribute reduct.

**Definition 8 .**

Let *S* = (*U*, *A*, *L*) be a multilabel decision table and *B*⊆*A*. If *η*_*B*_^*δ*^(*x*) = *η*_*A*_^*δ*^(*x*), for all *x* ∈ *U*, one says that *B* is a consistent *δ*-confidence set of *S*. If *B* is a consistent *δ*-confidence set and no proper subset of *B* is a consistent *δ*-confidence set, then *B* is called a *δ*-confidence reduct of *S*.

A *δ*-confidence reduct is the minimal set of condition attributes that preserves the invariances of the *δ*-confidence label function of all objects in *U*.

**Example 9 (continued from Example 6.).**

For the multilabel decision table *S* = (*U*, *A*, *L*) given by Table 1, we have

(11) η{a}0.6(x1)={l1,l2}≠ηA0.6(x1),η{b}0.6(x8)={l2,l3}≠ηA0.6(x8),η{c}0.6(x6)={l1}≠ηA0.6(x6),η{a,c}0.6(x1)={l2}≠ηA0.6(x1),η{b,c}0.6(x8)={l2,l3}≠ηA0.6(x8),η{a,b}0.6(x)=ηA0.6(x) for any  x∈U.

Therefore, we obtain the unique 0.6-confidence reduct: {*a*, *b*}.

Considering Example 3, however, we know that {*a*, *c*} and {*b*, *c*} are two positive region reducts for the same multilabel decision table. We think *δ*-confidence reduct is more appropriate for multilabel data than positive region reduct. This is because *δ*-confidence label function can more reasonably characterize the uncertainty implied among labels than the indiscernibility relation *R*_*L*_.

Note that the uncertainty characterized by *R*_*L*_ is also considered by the other existing attribute reduction methods. Therefore, for multilabel data, *δ*-confidence reduct has significant advantages when compared with existing attribute reduction methods.

### 4.2. Discernibility Matric of *δ*-Confidence Reduct

This section provides a discernibility matrice approach [12] to obtain all *δ*-confidence reducts. Firstly, we present the judgement theorem of consistent *δ*-confidence set.

**Theorem 10 (judgement theorem of consistent *δ*-confidence set).**

Let *S* = (*U*, *A*, *L*) be a multilabel decision table, *B*⊆*A* and *δ* ∈ [0,1]. Then the following conditions are equivalent:

1. *B* is a consistent *δ*-confidence set;
2. for any *x*, *y* ∈ *U*, if *η*_*A*_^*δ*^(*x*) ≠ *η*_*A*_^*δ*^(*y*), then [*x*]_*B*_∩[*y*]_*B*_ = *∅*.

**Proof**

(1)⇒(2). If there exist *x*, *y* ∈ *U* such that [*x*]_*B*_∩[*y*]_*B*_ ≠ *∅*, then [*x*]_*B*_ = [*y*]_*B*_. By Theorem 7(4), we have *η*_*B*_^*δ*^(*x*) = *η*_*B*_^*δ*^(*y*). Note that *B* is a consistent *δ*-confidence set; we have *η*_*B*_^*δ*^(*x*) = *η*_*A*_^*δ*^(*x*) and *η*_*B*_^*δ*^(*y*) = *η*_*A*_^*δ*^(*y*). Therefore *η*_*A*_^*δ*^(*x*) = *η*_*A*_^*δ*^(*y*).

(2)⇒(1). Since *B*⊆*A*, it is easy to verify that *I*([*x*]_*B*_) = {[*y*]_*A*_ : [*y*]_*A*_⊆[*x*]_*B*_} forms a partition of [*x*]_*B*_.

For any *x* ∈ *U*, if [*y*]_*A*_⊆[*x*]_*B*_, then [*x*]_*B*_∩[*y*]_*B*_ ≠ *∅*. By the assumption we obtain *η*_*A*_^*δ*^(*x*) = *η*_*A*_^*δ*^(*y*).

Let *l*_*i*_ ∈ *η*_*A*_^*δ*^(*x*). Then for all [*y*]_*A*_ ∈ *I*([*x*]_*B*_), we have *l*_*i*_ ∈ *η*_*A*_^*δ*^(*y*); that is to say, |[*y*]_*A*_∩*H*_*i*_|/|[*y*]_*A*_| ≥ *δ*.

Therefore we have that

(12) |[x]B∩Hi||[x]B|=∑{|[y]A∩Hi|:[y]A∈I([x]B)}|[x]B|=∑{|[y]A∩Hi||[y]A|·|[y]A||[x]B|:[y]A∈I([x]B)}⩾δ·∑{|[y]A||[x]B|:[y]A∈I([x]B)}=δ.

As a result, *l*_*i*_ ∈ *η*_*B*_^*δ*^(*x*).

On the other hand, we assume that *l*_*i*_ ∈ *η*_*B*_^*δ*^(*x*); however, *l*_*i*_ ∉ *η*_*A*_^*δ*^(*x*). For any [*y*]_*A*_ ∈ *I*([*x*]_*B*_), we have *η*_*A*_^*δ*^(*x*) = *η*_*A*_^*δ*^(*y*); hence *l*_*i*_ ∉ *η*_*A*_^*δ*^(*y*); that is to say, |[*y*]_*A*_∩*H*_*i*_|/|[*y*]_*A*_| < *δ*. Since [*x*]_*B*_ = ∪{[*y*]_*A*_ : [*y*]_*A*_ ∈ *I*([*x*]_*B*_)}, we have

(13) |[x]B∩Hi||[x]B|=∑{|[y]A∩Hi|:[y]A∈I([x]B)}|[x]B|=∑{|[y]A∩Hi||[y]A|·|[y]A||[x]B|:[y]A∈I([x]B)}<δ·∑{|[y]A||[x]B|:[y]A∈I([x]B)}=δ.

Therefore *l*_*i*_ ∉ *η*_*B*_^*δ*^(*x*), which is a contradiction.

Thus we conclude that *η*_*A*_^*δ*^(*x*) = *η*_*B*_^*δ*^(*x*) for any *x* ∈ *U*. According to Definition 8, we have that *B* is a consistent *δ*-confidence set.

Theorem 10 provides an approach to judge whether a subset of attributes is a consistent *δ*-confidence set in multilabel decision tables. Now we present a method for computing all *δ*-confidence reducts. First, we give the following notion.

**Definition 11 .**

Let *S* = (*U*, *A*, *L*) be a multilabel decision table and *U*/*R*_*A*_ = {*X*_1_, *X*_2_,…, *X*_*m*_}. One denotes

(14) $$\begin{array}{c} \Delta^{\delta }=\left\{\left({\left[x\right]}_{A},{\left[y\right]}_{A}\right):\eta_{A}^{\delta }\left(x\right)\neq \eta_{A}^{\delta }\left(y\right)\right\}. \end{array}$$

By *a*_*k*_(*X*_*i*_) the value of *a*_*k*_ with respect to the objects in *X*_*i*_. Define

(15) Mδ(Xi,Xj)={{ak∈A:ak(Xi)≠ak(Xj)},(Xi,Xj)∈Δδ;A,(Xi,Xj)∉Δδ.

Then *M* ^*δ*^(*X*_*i*_, *X*_*j*_) is called *δ*-confidence discernibility attribute sets. And *M* ^*δ*^ = (*M* ^*δ*^(*X*_*i*_, *X*_*j*_), *i*, *j* ≤ *m*) is called the *δ*-confidence discernibility matrix.

For the *δ*-confidence discernibility matrix, we have the following property.

**Theorem 12 .**

The discernibility matrix *M* ^*δ*^ = (*M* ^*δ*^(*X*_*i*_, *X*_*j*_), *i*, *j* ≤ *m*) satisfies the following properties:

1. *M* ^*δ*^ is a symmetric matrix; that is, for any *i*, *j* ≤ *m*, *M* ^*δ*^(*X*_*i*_, *X*_*j*_) = *M* ^*δ*^(*X*_*j*_, *X*_*i*_);
2. elements in the main diagonals are all *A*; that is, for any *i* ≤ *m*, *M* ^*δ*^(*X*_*i*_, *X*_*i*_) = *A*;
3. for any *i*, *s*, *j* ≤ *m*, *M* ^*δ*^(*X*_*i*_, *X*_*j*_)⊆*M* ^*δ*^(*X*_*i*_, *X*_*s*_) ∪ *M* ^*δ*^(*X*_*s*_, *X*_*j*_).

**Proof**

The proofs of (1) and (2) are straightforward. We only need to prove (3). If there exists *a*_*k*_ ∈ *A* such that *a*_*k*_ ∈ *M* ^*δ*^(*X*_*i*_, *X*_*j*_) but *a*_*k*_ ∉ *M* ^*δ*^(*X*_*i*_, *X*_*s*_) ∪ *M* ^*δ*^(*X*_*s*_, *X*_*j*_), that is, *a*_*k*_ ∉ *M* ^*δ*^(*X*_*i*_, *X*_*s*_) and *a*_*k*_ ∉ *M* ^*δ*^(*X*_*s*_, *X*_*j*_), then according to Definition 11, we have *a*_*k*_(*X*_*i*_) = *a*_*k*_(*X*_*s*_) and *a*_*k*_(*X*_*s*_) = *a*_*k*_(*X*_*j*_). Thus *a*_*k*_(*X*_*i*_) = *a*_*k*_(*X*_*j*_); that is, *a*_*k*_ ∉ *M* ^*δ*^(*X*_*i*_, *X*_*j*_), a contradiction.

In the following, we establish some connections between consistent *δ*-confidence set and discernibility matrix.

**Theorem 13 .**

Let *S* = (*U*, *A*, *L*) be a multilabel decision table, *B*⊆*A* and *δ* ∈ [0,1]. Then, *B* is a consistent *δ*-confidence set if and only if *B*∩*M* ^*δ*^(*X*_*i*_, *X*_*j*_) ≠ *∅* for all (*X*_*i*_, *X*_*j*_) ∈ Δ^*δ*^.

**Proof**

“⇒” For any (*X*_*i*_, *X*_*j*_) ∈ Δ^*δ*^, there exist *x*, *y* ∈ *U*, such that *X*_*i*_ = [*x*]_*A*_ and *X*_*j*_ = [*y*]_*A*_. From the definition of Δ^*δ*^, we have *η*_*A*_^*δ*^(*x*) ≠ *η*_*A*_^*δ*^(*y*). Since *B* is a consistent *δ*-confidence set, we have [*x*]_*B*_∩[*y*]_*B*_ = *∅* from Theorem 10. Therefore there exists *a*_*k*_ ∈ *B* such that *a*_*k*_(*x*) ≠ *a*_*k*_(*y*); that is, *a*_*k*_(*X*_*i*_) ≠ *a*_*k*_(*X*_*j*_). Hence *a*_*k*_ ∈ *M* ^*δ*^(*X*_*i*_, *X*_*j*_); that is, *B*∩*M* ^*δ*^(*X*_*i*_, *X*_*j*_) ≠ *∅*.

“⇐” Let (*X*_*i*_, *X*_*j*_) ∈ Δ^*δ*^. Since *B*∩*M* ^*δ*^(*X*_*i*_, *X*_*j*_) ≠ *∅*, for all (*X*_*i*_, *X*_*j*_) ∈ Δ^*δ*^, there exists *a*_*l*_ ∈ *B* such that *a*_*l*_ ∈ *M* ^*δ*^(*X*_*i*_, *X*_*j*_). Then we have *a*_*l*_(*X*_*i*_) ≠ *a*_*l*_(*X*_*j*_); that is, *a*_*l*_(*x*) ≠ *a*_*l*_(*y*) for [*x*]_*A*_ = *X*_*i*_ and [*y*]_*A*_ = *X*_*j*_. It means [*x*]_*B*_∩[*y*]_*B*_ = *∅*. We then conclude that if (*X*_*i*_, *X*_*j*_) ∈ Δ^*δ*^, that is, *η*_*A*_^*δ*^(*x*) ≠ *η*_*A*_^*δ*^(*y*), then [*x*]_*B*_∩[*y*]_*B*_ = *∅*. It follows from Theorem 10 that *B* is a consistent *δ*-confidence set.

Next we introduce the concept of discernibility function which helps us to compute *δ*-confidence reduct.

**Definition 14 .**

Let *S* = (*U*, *A*, *L*) be a multilabel decision table, let *δ* ∈ [0,1], and let *M* ^*δ*^ = (*M* ^*δ*^(*X*_*i*_, *X*_*j*_), *i*, *j* ≤ *m*) be the *δ*-confidence discernibility matrix, where *A* = {*a*_1_,…, *a*_*p*_}. A *δ*-confidence discernibility function *F*_*S*_^*δ*^ for a multilabel decision table *S* is a boolean function of *p* boolean variables ${\tilde{a}}_{1},\ldots ,{\tilde{a}}_{p}$ corresponding to the attributes *a*_1_,…, *a*_*p*_, respectively, and is defined as follows:

(16) FSδ(a~1,…,a~p)=⋀{⋁Mδ(Xi,Xj),i,j≤m}=⋀{⋁Mδ(Xi,Xj),(Xi,Xj)∈Δδ},

where ⋁*M* ^*δ*^(*X*_*i*_, *X*_*j*_) is the disjunction of all variables $\tilde{a}$ such that *a* ∈ *M* ^*δ*^(*X*_*i*_, *X*_*j*_).

In the sequel we will write *a*_*i*_ instead of ${\tilde{a}}_{i}$ when no confusion arises. Furthermore, according to related logical knowledge, we have the following theorem.

**Theorem 15 .**

Let *S* = (*U*, *A*, *L*) be a multilabel decision table. Then an attribute subset *B* of *A* is a *δ*-confidence reduct of *S* if and only if ∧*B* is a prime implicant of *S*.

Theorem 15 provides a discernibility matrix based method to compute all *δ*-confidence reducts. The following example illustrates the validity of the approach.

**Example 16 .**

Consider the multilabel decision table given by Table 1. We have *U*/*R*_*A*_ = {*X*_1_,…, *X*_5_}, where

(17) X1={x1,x3},X2={x2},X3={x4,x5,x7},X4={x6},X5={x8,x9}.

According to the calculation results of *η*_*A*_^0.6^(*x*) in Example 6, we have

(18) Δ0.6={(X1,X3),(X1,X4),(X1,X5),(X2,X3),(X2,X4),(X2,X5),(X3,X4),(X3,X5),(X4,X5)}.

Note that *η*_*A*_^0.6^(*x*_1_) = *η*_*A*_^0.6^(*x*_2_) = *η*_*A*_^0.6^(*x*_3_). Therefore (*X*_1_, *X*_2_) ∉ Δ^0.6^.

We can calculate the *δ*-confidence discernibility matrix shown in Table 2.

Consequently, we have

(19) F=(a∨b∨c)∧(a∨b)∧(a)∧(b)∧(b∨c)=a∧b.

By Theorem 15 we derive that {*a*, *b*} is the unique 0.6-confidence reduct which accords with the results in Example 9.

## 5. Conclusion

The *δ*-confidence reduct presented in this paper is an attribute reduction method designed for multilabel decision tables. Compared with the existing attribute reduction methods, the *δ*-confidence reduct accurately characterizes uncertainty implied among labels; thus it is more appropriate for multilabel data. Moreover we proposed the corresponding discernibility matrix based method to compute *δ*-confidence reduct, which is significant in both the theoretic and applied perspectives. In further research, the property of *δ*-confidence reduct and corresponding heuristic algorithm will be considered.

## Figures and Tables

**Table 1:** A multilabel decision table.

*U*	*a*	*b*	*c*	*l*_1_	*l*_2_	*l*_3_
*x*_1_	2	1	0	1	0	0
*x*_2_	2	1	1	1	0	0
*x*_3_	2	1	0	1	1	0
*x*_4_	1	3	0	1	1	1
*x*_5_	1	3	0	0	1	0
*x*_6_	3	2	1	1	1	1
*x*_7_	1	3	0	0	1	1
*x*_8_	2	3	0	0	1	0
*x*_9_	2	3	0	0	1	1

**Table 2:** The *δ*-confidence discernibility matrix *M*.

	*X*_1_	*X*_2_	*X*_3_	*X*_4_	*X*_5_
*X*_1_					
*X*_2_	*a*, *b*, *c*				
*X*_3_	*a*, *b*	*a*, *b*, *c*			
*X*_4_	*a*, *b*, *c*	*a*, *b*	*a*, *b*, *c*		
*X*_5_	*b*	*b*, *c*	*a*	*a*, *b*, *c*	
